# The spatio-temporal properties of calcium transients in hippocampal pyramidal neurons *in vitro*

**DOI:** 10.3389/fncel.2022.1054950

**Published:** 2022-12-14

**Authors:** Vyacheslav M. Shkryl

**Affiliations:** Department of Biophysics of Ion Channels, Bogomoletz Institute of Physiology, NAS of Ukraine, Kyiv, Ukraine

**Keywords:** hippocampal neuronal culture, calcium homeostasis, ryanodine receptors, caffeine, ratiometric method, Fura-2

## Abstract

The spatio-temporal properties of calcium signals were studied in cultured pyramidal neurons of the hippocampus using two-dimensional fluorescence microscopy and ratiometric dye Fura-2. Depolarization-induced Ca^2+^ transients revealed an asynchronous delayed increase in free Ca^2+^ concentration. We found that the level of free resting calcium in the cell nucleus is significantly lower compared to the soma, sub-membrane, and dendritic tree regions. Calcium release from the endoplasmic reticulum under the action of several stimuli (field stimulation, high K^+^ levels, and caffeine) occurs in all areas studied. Under depolarization, calcium signals developed faster in the dendrites than in other areas, while their amplitude was significantly lower since larger and slower responses inside the soma. The peak value of the calcium response to the application of 10 mM caffeine, ryanodine receptors (RyRs) agonist, does not differ in the sub-membrane zone, central region, and nucleus but significantly decreases in the dendrites. In the presence of caffeine, the delay of Ca^2+^ signals between various areas under depolarization significantly declined. Thirty percentage of the peak amplitude of Ca^2+^ transients at prolonged electric field stimulation corresponded to calcium release from the ER store by RyRs, while short-term stimulation did not depend on them. 20 μM dantrolene, RyRs inhibitor, significantly reduces Ca^2+^ transient under high K^+^ levels depolarization of the neuron. RyRs-mediated enhancement of the Ca^2+^ signal is more pronounced in the central part and nucleus compared to the sub-membrane or dendrites regions of the neuron. In summary, using the ratiometric imaging allowed us to obtain additional information about the involvement of RyRs in the intracellular dynamics of Ca^2+^ signals induced by depolarization or electrical stimulation train, with an underlying change in Ca^2+^ concentration in various regions of interest in hippocampal pyramidal neurons.

## Introduction

The calcium ion (Ca^2+^) is a common second messenger that regulates many physiological pathways such as secretion, fertilization, gene transcription, and apoptosis (Pozzan et al., 1994; Berridge et al., 2000). Calcium signaling in excitable cells consists of several mechanisms. In the first phase, Ca^2+^ influx across the plasma membrane after the opening of voltage-gated calcium channels (VGCCs) as a result of action potential (AP) depolarization or activation of ligand-gated channels (Berridge, 1998; Augustine et al., 2003; Bloodgood and Sabatini, 2007). Then Ca^2+^ can be released from the endoplasmic reticulum (ER) or sarcoplasmic reticulum (SR) through 1,4,5-Inositol triphosphate receptors (IP_3_Rs) and/or ryanodine receptors (RyRs) expressed on the ER (or SR) membrane (Berridge, 1998, 2009; Verkhratsky, 2002; Clapham, 2007; Khakh and McCarthy, 2015).

The release of Ca^2+^ from the SR store amplified Ca^2+^ signaling and regulates a variety of cellular processes, including excitation-contraction coupling in skeletal muscle (Endo et al., 1970; Ford and Podolsky, 1970) and cardiac myocytes (Fabiato, 1983; Bers, 2002), and from the ER store calcium oscillation and gene expression in many cell types (Thorn et al., 1993; Hardingham et al., 2001), neuronal plasticity (Frenguelli and Malinow, 1996; Rose and Konnerth, 2001) and other processes. The ER is an essential intracellular organelle—one of the main functions of which is the storage of intracellular Ca^2+^. The entry of calcium ions through VGCCs activates RyRs *via* a calcium-mediated process that obtained, at present, the generally known designation “calcium-induced calcium release,” CICR (Fabiato, 1983).

In most CNS neurons, free intracellular calcium concentration ([Ca^2+^]_i_) changes following the opening of VGCCs (Berridge, 1998; Augustine et al., 2003; Bloodgood and Sabatini, 2007) and then is buffered with some proteins like calmodulin, calreticulin, parvalbumin, calbindin-D28k. In addition, ATP binds significant amounts of Ca^2+^ ions (Zhou and Neher, 1993; Shkryl et al., 2012a). As in other cell types, in neurons, Ca^2+^ is released from the ER through IP_3_Rs and/or RyRs expressed on the ER membrane, which regulates a myriad of physiological and pathophysiological processes in it (Verkhratsky and Kettenmann, 1996; Berridge, 1998, 2002, Berridge, 2009; Verkhratsky, 2002, 2005; Clapham, 2007; Khakh and McCarthy, 2015; Shkryl, 2017). The binding of Ca^2+^ to RyRs activates these channels allowing Ca^2+^ to release from the ER into the cytosol (Kuba, 1994). Activation of RyRs is linked to L-type VDCCs either *via* Ca^2+^ influx in cardiac myocytes or by direct voltage-dependent mechanical coupling in skeletal muscle (Pozzan et al., 1994; Niggli, 1999). The latter type of coupling has also been observed in neuronal cells (Chavis et al., 1996; De Crescenzo et al., 2004).

RyRs in peripheral and central neurons may amplify and prolong incoming Ca^2+^ signals *via* CICR (Holliday et al., 1991; Llano et al., 1994; Kano et al., 1995). Some studies have shown that depleting or blocking the ryanodine-sensitive Ca^2+^ stores did not significantly alter the amplitude and waveform of depolarization-induced Ca^2+^ transients and did not contribute significantly to depolarization-induced Ca^2+^ transients evoked by low-frequency activity (Garaschuk et al., 1997). However, it established that the soma of pyramidal neurons provides a reticular network of the ER and extends throughout dendrites and the entire length of the axon, including presynaptic boutons (de Juan-Sanz et al., 2017). In addition, repetitive tetanic synaptic stimulation of CA1 pyramidal cells in a slice preparation has been reported to induce Ca^2+^ release from dendritic or presynaptic ryanodine-sensitive Ca^2+^ stores (Alford et al., 1993; Tran and Stricker, 2021).

There are broadly selective drugs that are able to activate or inhibit RyRs in myocytes or neurons. The most well-known are caffeine, ryanodine, 4-chloro-m-cresol, dantrolene, ruthenium red, tetracaine, and procaine (Viero et al., 2012). Caffeine is a RyRs agonist and has been used as a pharmacological tool to study ryanodine receptor-mediated Ca^2+^ release from intracellular stores (Kong et al., 2008; Porta et al., 2011). Caffeine sensitizes RyRs to Ca^2+^ and promotes ER Ca^2+^ release at basal cytosolic Ca^2+^ levels. Another drug is RyRs-inhibitor dantrolene (Fruen et al., 1997; Fill and Copello, 2002) which in the concentration of 10 μM reduced RyRs channel open probability by ~50% (Diszházi et al., 2019).

Information about the involvement of RyRs in calcium signaling in neurons is controversial, especially about where release occurs and how it interacts with the Ca^2+^ signal during short or long-term stimulation. This study aimed to obtain additional information on the involvement of ryanodine receptors in neuronal calcium signaling. Spatio-temporal properties of these signals were studied during multiple stimuli: field stimulation, high K^+^, and caffeine and dantrolene at cultured rat hippocampal CA1 pyramidal neurons. We use caffeine and dantrolene to reveal RyRs. Changes in free calcium concentrations inside the cell were determined using two-dimensional fluorescence microscopy based on a charge-coupled device camera and ratiometric dye Fura-2 with dye excitation at wavelengths 340–380 nm. We analyzed two-dimensional fluorescent images of neurons spatially with temporal profiles from different areas of it. For such analysis were selected: the nucleus, sub-membrane region, central space inside of a cell, and the dendritic tree. This technique allows us to simultaneously determine the change in Ca^2+^ signals in the different regions of the neurons.

## Materials and Methods

All experimental procedures were performed following ethical principles of the European Convention for the protection of vertebrate animals used for experimental and other scientific purposes (86/609/EEC; European convention, Strasburg, 1986) and were approved by the local Animal Ethics Committee of the Bogomoletz Institute of Physiology (Kyiv, Ukraine). All efforts were made to minimize the number and suffering of animals used. All experiments were performed on cultured hippocampal neurons obtained from newborn Wistar rats. Primary cultures were prepared as previously reported (Shkryl et al., 1999). Ca^2+^ imaging studies were carried out within 10–14 days of cultivation. A cover glass with cells was placed in an extracellular solution (ES) containing in mM: NaCl—140.0; KCl—2.0; CaCl_2_—2.0; MgCl_2_—2.0; HEPES—10.0; pH = 7.4. All chemicals were obtained from Sigma-Aldrich (St. Louis, MO).

The cells were loaded with 5 μM Fura-2 acetoxymethyl ester (Fura-2 AM) for 30 min at 37°C and 20 min for de-esterification of the dye. The values of R_min_ and R_max_ amounted to 0.44 and 9.44; *F*_380_(min) = 20.7 and *F*_380_(max) = 169.4; *K_d_*(Fura-2) = 224 nM (Shkryl, 2020). The coverslip with neurons was washed and placed in an experimental chamber with two platinum electrodes used for the electric field stimulation (EFS). The stimulation was carried out according to the standard protocol by lowering two parallel platinum electrodes (20÷25 mm apart) into the chamber and passing current pulses between them (Jacobs and Meyer, 1997; Shkryl et al., 2012b). Ca^2+^ transients were also induced chemically by applying a depolarizing solution that contained 50.0 mM KCl substituting the respective amount of NaCl in ES. We used a computer-controlled perfusion system synchronized with data acquisition software.

In our study, we used a CCD camera (Olympus XM10) mounted on an Olympus IX71 inverted microscope equipped with an Olympus LUCPlanFFN 20×/0.45 lens and MT10 illumination system that included a filter wheel exchanger (340–380 nm) and 150 W xenon arc burner. For the data collection, we use Cell M software (Olympus, Japan). The acquisition speed was 30 Hz for one wave and 1.3 Hz for ratiometric use. For further data analysis, the ratio of 340–380 nm fluorescence intensity (ratio; F_340_/F_380_) was calculated. This is done according to the protocol described by Shkryl (Shkryl, 2020) and subtracts the background level calculated outside of cells. Dynamic changes in the ratio index assess changes in the level of free calcium.

Data analysis was performed using the IDL programming environment (ITT Visual Information Solutions). In recorded images with excitation of 340–380 nm lights, the regions of interests (ROIs) were selected. All experiments were performed at room temperature (22°C–25°C). Data are presented as mean ± SEM.

## Results

In most neurons, the intracellular concentration of free calcium in the resting state is around 75 nM. This index can increase to 10 μM upon intense electrical activity of cells (Berridge et al., 2000). In particular, the calcium level in the dendrites could increase to 5 μM during the development of synaptically induced calcium waves (Pozzo Miller et al., 1996; Larkum et al., 2003). Information on the spatial distribution of the respective calcium channels and receptors is limited; however, it is clear that the molecular mechanisms and patterns of Ca^2+^ release in different regions of different neurons differ significantly (Verkhratsky, 2005). To examine changes in the intracellular calcium level in cultured pyramidal hippocampal neurons, we used 2D x-y-t ratiometric fluorescent Ca^2+^ measurements and Ca^2+^-sensitive dye Fura-2, AM. In this series of experiments, the cells constantly perfused with ES were stimulated by EFS and by applying 50 mM KCl or 10 mM caffeine solutions.

### Ca^2+^ responses in different regions of pyramidal hippocampal neuron

**Figure 1:**
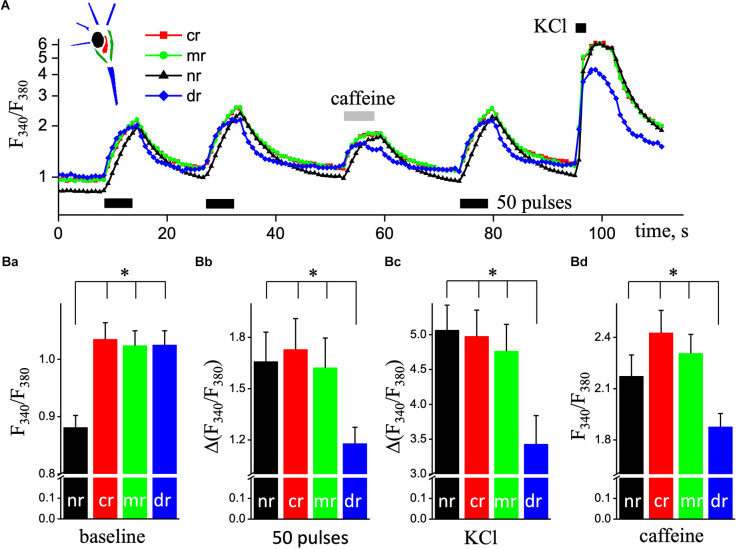
Ca^2+^ responses in different ROIs of pyramidal hippocampal neurons triggered by electrical field stimulation and application of depolarizing and caffeine solutions. **(A)** Transient increases in [Ca^2+^]_i_ are triggered by two series of 50 pulses of EFS (black rectangular, 9 Hz) followed by the application of caffeine (caffeine, light gray rectangular; 10 mM and 5 s) and a series of 50 pulses of EFS and depolarizing solution with 50 mM KCL (KCl, black square, 5 s). Ca^2+^ responses were recorded in the central space (cr; red squares line), sub-membrane (mr; green circles line), dendritic tree (dr; blue diamond’s line), and nucleus (nr; black triangles lines) regions of cultured hippocampal neurons loaded with the fluorescent Ca^2+^ indicator Fura-2. Ratios (F_340_/F_380_) are presented in natural logarithmic scale. The top left corner represents a schema of neurons’ area selection. The bottom panel **(B)** shows quantified responses for four studied regions in pyramidal neurons. **(Ba)** Mean value of basal Ca^2+^ levels before any stimulus; part **(Bb)** is the mean values of the amplitudes of second Ca^2+^ transients caused by EFS; part **(Bc)** is the mean values of the amplitudes of Ca^2+^ transients evoked by depolarizing solution; part **(Bd)** is the mean values of Ca^2+^ peaks evoked by 5 s application of 10 mM caffeine, as an agonist of RyRs. The black bars are data for the nucleus (nr); the red bars are data in the central space (cr); the green bars are sub-membrane regions (mr), and the blue bars are dendritic trees of neurons (dr). Results are mean ± S.E. of 30 individual cells from 10 different experiments. *P < 0.05.

We analyzed Ca^2+^ signals in hippocampal pyramidal neurons in the sub-membrane, central, dendritic, and nuclear regions. Figure 1A illustrates representative data traces of the Fura-2 ratio (F_340_/F_380_; Ca^2+^ signals) increases induced by two series of 50 pulses of EFS (used to load the ER store) and followed by the application of 10 mM caffeine for 5 s to induce Ca^2+^ release from the ER stores by ryanodine receptors (RyRs). An additional EFS and 5-s application of the depolarizing solution were applied when the restoration of calcium signals to the resting level. 2D imaging of Ca^2+^ signals reveals an asynchronous delayed rise of free Ca^2+^ concentration in the nuclear compared to the sub-membrane, central or dendritic regions (Figure 1A). Statistical data are presented in the bottom plane of Figure 1B.

The value of basal free Ca^2+^ was significantly reduced in the nuclear area of the neuron, less by 15% compared to the central region (61 nM vs. 85 nM, *n* = 30; *P* < 0.001; Figure 1Ba). Ca^2+^ signal in the dendritic region in response to stimulation appeared faster compared to other regions (detailed below) but had the smallest amplitude during EFS, KCl depolarization, and caffeine application. The amplitude of Ca^2+^ transient in the dendritic tree of pyramidal-like neurons in response to EFS was 1.18 ± 0.09 (207 nM, *n* = 19), which was significantly smaller compared to sub-membrane—1.62 ± 0.17 (303 nM, *n* = 19; *P* < 0.05), central—1.73 ± 0.18 (329 nM, *n* = 19; *P* < 0.01), and nuclear—1.66 ± 0.17 (300 nM, *n* = 19; *P* < 0.05) regions of the cell (Figure 1Bb). In the dendritic tree, the amplitude of Ca^2+^ transient caused by KCl depolarization was also significantly reduced, 3.43 ± 0.41 (0.87 μM, *n* = 24) respectively to sub-membrane—4.76 ± 0.39 (1.65 μM, *n* = 24; *P* < 0.05), central—4.98 ± 0.38 (1.84 μM, *n* = 24; *P* < 0.01), and nuclear—5.06 ± 0.39 (1.81 μM, *n* = 24; *P* < 0.01) regions of studied neurons (Figure 1Bc). As shown in Figure 1Bd; the peak values of calcium signals induced by 10 mM caffeine (as Ca^2+^ release from the ER) were significantly reduced in the dendritic tree region to 1.88 ± 0.08 (225 nM, *n* = 19) from 2.31 ± 0.11 (310 nM, *n* = 19, *P* < 0.01) in sub-membrane, central—2.43 ± 0.13 (336 nM, *n* = 19; *P* < 0.001) and nuclear—2.17 ± 0.12 (283 nM, *n* = 19; *P* < 0.05) regions of pyramidal neurons.

### Fast recording of Ca^2+^ transients

**Figure 2:**
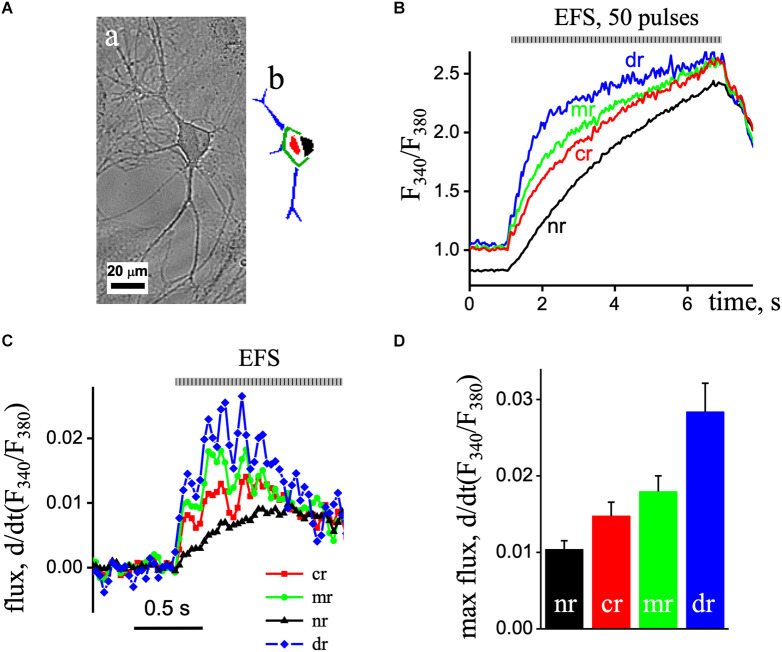
Fast recording of EPS-induced Ca^2+^ transients in a pyramidal-like hippocampal neuron. **(Aa)** The image of the cell obtained by direct illumination. **(Ab)** Different ROIs of the neuron are colored. **(B)** The imaged ratio of F340/F380 of Fura-2 loaded neuron with 50 EPS was separated as time profiles in the dendritic tree (blue line; dr), sub-membrane (green line, mr), central area (red line; cr), and nuclear (black line; nr) regions of the neuron. **(C)** d(F340/F380)/dt (s^−1^), the first derivative of the ratio of the Ca^2+^ signal obtained in the nuclear (black triangles line), central area (red squares line), and sub-membrane (green circles line) regions of the neuron. The flux data were derived from the data presented in part **(B)**. **(D)** Mean value of the maximal flux rate obtained in the nuclear (black bar), central area (red bar), and sub-membrane (green bar) regions of the neuron.

In the next series of experiments, we investigated the spatio-temporal organization of Ca^2+^ transients in the various areas of pyramidal-like neurons recorded at 30 Hz frequencies (Figure 2A). We recorded simultaneously the Ca^2+^ transients induced by ESP stimulation at excitation wavelengths 340–380 nm, which were synchronized with EFS, separated in time. The first recorded Ca^2+^ transient was with excitation wavelength at 340 nm, and then a 2 min delay produced an additional record at 380 nm with the same setting and conditions that create the F_340_/F_380_ ratio signal (Figure 2B).

Fast 2D recording of Ca^2+^ transients under depolarization revealed an asynchronous delayed rise of free Ca^2+^ concentration. Thus, EFS caused an increase in Ca^2+^ signals that first appeared in the dendrites, then in the sub-membrane, central, and finally in the nuclear area of cells. Figure 2C shows the first derivative of the [Ca^2+^]_i_ signal [d(F340/F380)/dt] representing the underlying Ca^2+^ flux. The Ca^2+^ flux in the sub-membrane and central regions appeared with increasing and decreasing steps, representing the EFS-induced calcium transients. The maximum flux in the sub-membrane and central regions was observed 0.2 s after initiation and decreased with time. In the nuclear region of the neuron, the flux was delayed and reached its maximum value 0.6 s after the onset with reduced amplitude compared to other regions. The maximal flux rate was in the dendritic tree region (0.028 ± 0.004, *n* = 8) and reduced in the sub-membrane (0.018 ± 0.002, *n* = 8) and central (0.015 ± 0.004, *n* = 8), and minimal in nuclear aria (0.010 ± 0.001, *n* = 8) of the neuron (Figure 2D).

**Figure 3:**
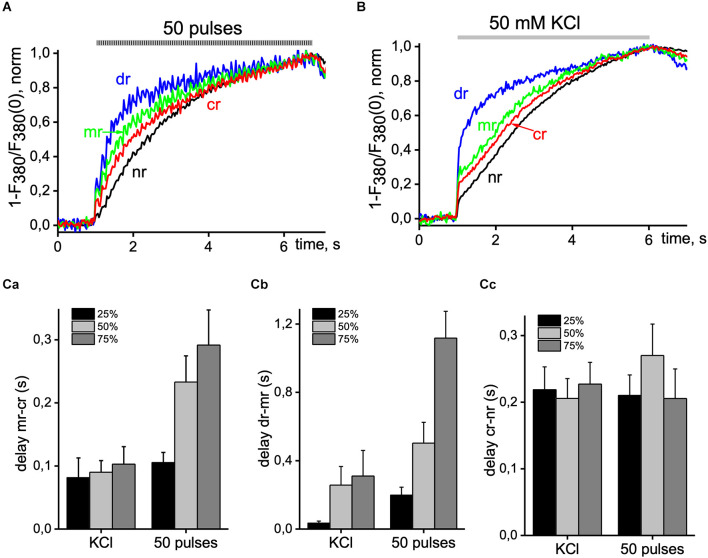
Delay of Ca^2+^ signals between distinct areas of pyramidal neurons. Normalized Ca^2+^ transients were recorded from the central (cr; red line), sub-membrane (mr; green line), nuclear (nr; black line), and dendritic tree (dr; blue line) area of the cell induced by 50 EFS pulses at 9 Hz **(A)** and 5 s application of 50 mM KCl solution **(B)**. **(C)** Summary histogram of delays between sub-membrane and central areas (mr-cr; part **a**); dendritic tree and sub-membrane areas (dr-mr; part **b**); central and nuclear areas (cr-nr; part **c**) measured at 25% (black bar), 50% (light gray bar) and 75% (dark gray bar) of maximal amplitude value with 50 pulses EFS and application of depolarizing KCl solution. Data were obtained in seven neurons in four different experiments. Ca^2+^ signals were recorded with 380 nm excitation wavelength at 33 ms per image.

To show more detailed information about the inhomogeneity of Ca^2+^ signals in hippocampal pyramidal neurons, we studied the latency of Ca^2+^ increases for different regions of cells in one wavelength excitation mode (380 nm, 30 Hz). Figure 3 shows the dynamics of [Ca^2+^]_i_ increases caused by 50 pulses of EFS (3A) and 50 mM KCl solution (3B) normalized to the maximal amplitude of Ca^2+^ transients in various ROIs of the neuron. When recording in x-y-t mode (limited to 30 Hz), Ca^2+^ signals were insufficient to represent the initial phase of the Ca^2+^ signal, which was more evident during KCl-induced transients. However, we find signal delays between different ROIs at 25%, 50%, and 75% of the maximal amplitude. It is in the sub-membrane, and central regions were more pronounced and progressed during the EFS-induced Ca^2+^ transient and less during KCl depolarization, and this parameter of the difference between the dendritic tree and the sub-membrane region of interest increased up to 1 s. In the central and nuclear regions, the delay was almost independent of the type of stimulus and was around 0.2 s. Statistical data are represented in the summarized diagram in part 3C.

### RyRs mediated Ca^2+^ signal

As is well known, Ca^2+^ release from the ER occurs under conditions of the opening of the IP_3_Rs and RyRs (Rizzuto and Pozzan, 2006). The IP_3_Rs-mediated release of calcium from the dendrites is initiated most frequently by the action of neurotransmitters, like glutamate (Niswender and Conn, 2010), while RyRs activate by an increase of Ca^2+^ concentration in the cytosol (Shkryl and Blatter, 2013; Blatter, 2017) *via* calcium-mediated activation RyRs through CICR (Fabiato, 1983). In the following experiments, we tested the functioning of the ER stores in hippocampal pyramidal neurons. For this purpose, we activated Ca^2+^ release from the ER by applications of the RyR agonist caffeine, which often revealed an increase in cytosolic Ca^2+^ concentrations induced by caffeine. We use ESP to load the ER store.

**Figure 4:**
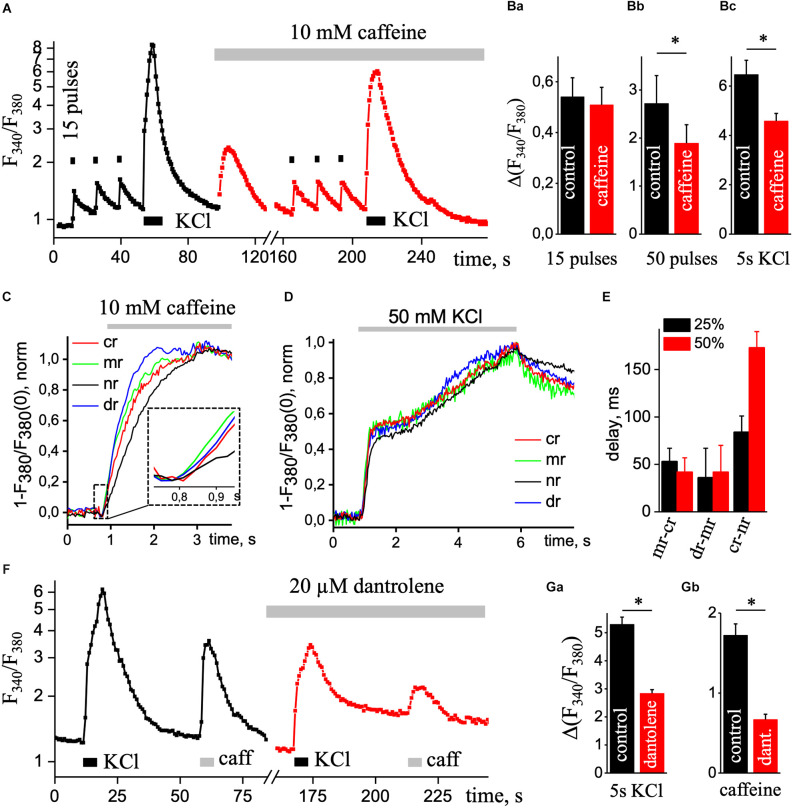
Ca^2+^ transients under the presence of caffeine. **(A)** Transient increases in [Ca^2+^]_i_ are triggered by three series of 15 pulses of EFS (black square; 1 s) followed by application of KCl depolarizing solution (KCl, black rectangle, 5 s) in the control condition (black square line) and repeated stimulations with continues presence of 10 mM caffeine (caffeine; red square line) to eliminate calcium release from the ER. Ca^2+^ responses were recorded in the soma without the nucleus region. The ratio (F_340_/F_340_) is presented on a natural logarithmic scale. **(B)** Mean values of the amplitude of the second Ca^2+^ transient caused by 15 **(a)** or 50 **(b)** pulses EFS and Ca^2+^ transient evoked by KCl solution **(c)** in control and presence of 10 mM caffeine. Normalized Ca^2+^ transients were recorded using one wavelength protocol (33 ms per image and 380 nm excitation wavelength) at different ROIs induced by the application of 10 mM caffeine **(C)** or application of KCl depolarizing solution **(D)**. A dashed line in part **(C)** contains an insert of the initial phase of caffeine-induced Ca^2+^ increase. ROIs were selected in central (cr, red line), sub-membrane (mr, green line), nuclear (nr, black line), and dendritic tree (dr, blue line) areas of the cell. **(E)** Diagram of delays between sub-membrane and central (mr-cr); dendritic tree and sub-membrane (dr-mr); central and nuclear (cr-nr) areas measured at 25% (black bar) and 50% (red bar) of the maximal amplitude in response to KCl depolarizing solution. **(F)** Transient increases in [Ca^2+^]_i_ are triggered by the application of KCl depolarizing solution (KCl, black rectangle, 5 s) and caffeine (caffeine, light gray rectangular; 10 mM and 5 s) in the control condition (black square line) and repeated stimulations with continues presence of 20 μM dantrolene (red square line) to reduce calcium release from the ER. Ca^2+^ responses were recorded in the soma without the nucleus region. The ratio (F_340_/F_340_) is presented on a natural logarithmic scale. Mean value of the amplitude of Ca^2+^ transients evoked by depolarizing solution **(Ga)** and 5 s application of 10 mM caffeine **(Gb)**. Results are mean ± S.E., *P < 0.05.

Figure 4A shows a superimposed record that demonstrates changes in Ca^2+^ signal, recorded from a neuron under control condition (black trace) and in the presence of 10 mM caffeine (red trace). In control, the basal level of free calcium was 1.06 ± 0.03 and 1.26 ± 0.05 in the presence of caffeine. The peak amplitude of Ca^2+^ transients induced by depolarizing solution significantly decreased from 6.47 ± 0.57 (in the control) to 4.58 ± 0.31 under the application of 10 mM caffeine (Figure 4Bc; *n* = 12, *P* < 0.05). Under electrical field stimulation changing the amplitude of the caffeine depend on the duration of the stimulation. The peak amplitude of the Ca^2+^ signal induced by short (15 pulses, 9 Hz) EFS did not change appreciably, 0.54 ± 0.08 compared to 0.51 ± 0.07 (Figure 4Ba; *n* = 13). However, by a long stimulus (50 pulses, 9 Hz), this parameter significantly decreases from 2.72 ± 0.58 to 1.89 ± 0.38 (Figure 4Bb; *n* = 6, *P* < 0.05) with caffeine.

The short electrical stimulation (15 pulses, 1 s) does not alter the amplitude of Ca^2+^ transients under caffeine, but the Ca^2+^ response significantly decreased under the depolarization solution or long EFS. Thus, with prolonged stimulation of the neuron, Ca^2+^ release appeared from the ER by RyRs. As we can see from normalized caffeine-induced Ca^2+^ transients (Figure 4C), a rise in signals appeared in all regions of hippocampal pyramidal neurons, but in the center and nuclear, Ca^2+^ signals were more delayed (37.5 ± 7.5 ms, *n* = 4) than in sub-membrane zone and dendritic tree. Figure 4D shows the dynamics of increases in [Ca^2+^]_i_ caused by 5 s application of KCl solution normalized to the maximal amplitude of the signal in various regions of interest of the neuron. Figure 4E shows the delay in different ROIs at 25% and 50% of the maximal amplitude. Under caffeine, the delay between sub-membrane and central, dendritic tree, central and nuclear regions was significantly reduced compared to control.

Additionally, we used 20 μM dantrolene to block RyRs and record high KCl-induced Ca^2+^ transients. Figure 4F shows superimposed record that demonstrates changes in Ca^2+^ signal, recorded from a neuron under control condition (black square line) and in the presence of 20 μM dantrolene (red square line). In control, the basal level of free calcium was 1.23 ± 0.03 and 1.10 ± 0.03 in the presence of dantrolene. The amplitude value of depolarizing solution-induced Ca^2+^ transients under 20 μM dantrolene significantly decreased from 5.30 ± 0.26 to 2.84 ± 0.13 (Figure 4Ga; *n* = 29, *P* < 0.001). The ER load with the presence of dantrolene was significantly decreased to 0.682 ± 0.06 from 1.72 ± 0.14 (Figure 4Gb; *n* = 28, *P* < 0.01). It further confirms the involvement of RyRs in Ca^2+^ signaling in cultured hippocampal pyramidal neurons.

**Figure 5:**
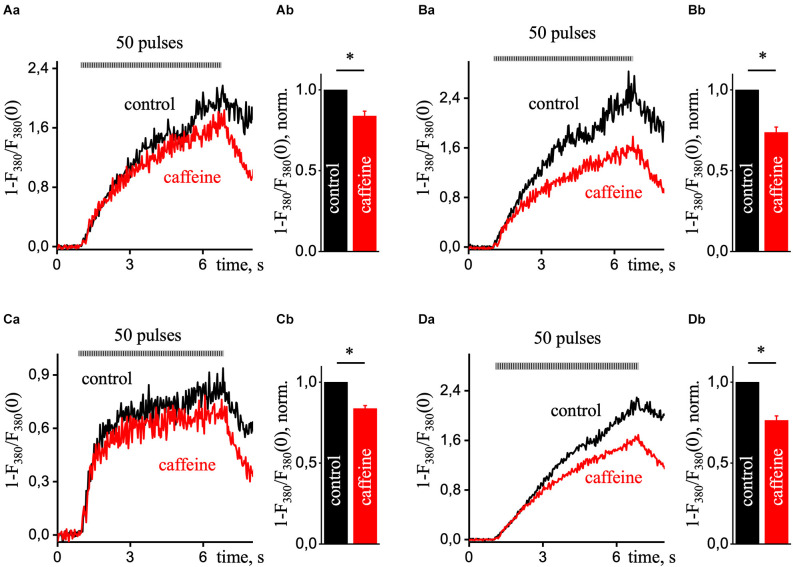
Ca^2+^ transients during prolonged electrical field stimulation in the presence of caffeine in various ROIs of hippocampal pyramidal neurons. A transient increase in intracellular [Ca^2+^]_i_ is induced by a series of 50 EFS pulses (9 Hz) under control conditions (black line) and in the presence of 10 mM caffeine (red line; which excludes calcium release from the ER). Each graph in part **(a)** presented Ca^2+^ responses that were recorded in the sub-membrane **(A)**, central **(B)**, dendritic tree **(C)**, and nucleus **(D)** of the cell. Normalized Ca^2+^ transients were recorded using a single wavelength protocol (33 ms per image and 380 nm excitation wavelength). Part **(b)** of each panel presents control-normalized statistics of maximal amplitude in the presence of caffeine in response to ESP. Results are mean ± S.E. *P < 0.05.

Figure 5 shows representative recordings of calcium transients induced by 50 pulses of EFS in control (black line) and the presence of 10 mM caffeine (red line) in different regions of the pyramidal neuron. The calcium signals in control and caffeine had a substantial difference at the center (Figure 5B), although there was also a decrease in signal with caffeine in the sub-membrane (Figure 5A) and dendritic tree regions (Figure 5C) were less pronounced but significant. Data were obtained from six different experiments and compared using a paired sample *t*-test. Also, the difference between these signals in the nucleus (Figure 5D) was somewhat less than in the central region. It is probably due to the passive enhancement of the calcium signal by releasing Ca^2+^ from the center or neighboring ERs into the nucleus. The RyR-mediated increase in the Ca^2+^ signal presented in the periphery, the dendritic tree, and the center of the hippocampal pyramidal neuron.

## Discussion

In this study, we investigated the behavior of Ca^2+^ signals in response to electrical stimulation, KCl-induced depolarization, and caffeine treatment in cultured hippocampal pyramidal neurons with a 2D imaging mode of ratiometric fluorescent microscopy.

A neuron is an excitable cell, and a fundamental principle in neuronal signal processing is the transduction of short electrical signals at the membrane into biochemical responses, resulting in longer-lasting changes in the neuronal structural and functional state (Johenning et al., 2015). The neuronal plasma membrane with channels, receptors, and pumps mediates the electrical signal propagation; the ER membrane can participate in the passive and active calcium-based signal propagation mainly along the dendritic length and soma from peripheries to the center (Shkryl, 2017; Ashhad and Narayanan, 2019). Action potentials generate widespread increases in calcium concentration inside axons and presynaptic terminals, and then they back-propagate over large regions of the dendrites (Ross, 2012). The present study shows that the level of free calcium in the resting state inside the nucleus is significantly lower compared to the center of the cell and delayed respectively to other regions of hippocampal pyramidal neurons. Fedorenko and Marchenko (2014) have shown that the nuclear membrane of hippocampal CA1 pyramidal neurons was enriched in functional inositol IP_3_Rs localized in the inner nuclear membrane and is specialized to release Ca^2+^ into the nucleoplasm, which may amplify Ca^2+^ signals entering the nucleus from the cytoplasm. Our study shows that nuclear Ca^2+^ flux had the lowest amplitude compared to other studied regions. During applications of caffeine, the nuclear Ca^2+^ signal was suspended compared to the sub-membrane but appeared at the same time as the central area with almost no delay.

Localized RyR-mediated events of Ca^2+^ release occur in the soma and proximal dendrites of hippocampal pyramidal neurons in culture and acute slices (Koizumi et al., 1999; Berrout and Isokawa, 2009; Manita and Ross, 2009; Miyazaki et al., 2012). The endoplasmic reticulum localized close to the plasmalemma membrane (PM) makes a membrane domain called ER-PM junctions (EPJs). By electron microscopy, the ER at many neuronal EPJs appears as a micron-diameter, flattened vesicle less than 10 nm from the PM, a structure also called a “subsurface cistern” (Rosenbluth, 1962; Tao-Cheng, 2018). EPJs are abundant in neuronal soma (Wu et al., 2017), and neuronal soma has prominent VGCCs—and RyRs-mediated CICR (Friel and Tsien, 1992; Isokawa and Alger, 2006; Berrout and Isokawa, 2009). Our study shows that the release of calcium from the endoplasmic reticulum occurred in all regions of the neuron; the peak value of Ca^2+^ response to caffeine applications did not differ at the sub-membrane, center, and nucleus but significantly decreased in the dendrites.

Ryanodine-sensitive Ca^2+^ stores in different central neurons in the rat (including CA1 hippocampal neurons) were reported to be empty at rest and to accumulate Ca^2+^ only after its entry *via* voltage-gated Ca^2+^ channels in the plasmalemma (Brorson et al., 1991; Shmigol et al., 1994; Garaschuk et al., 1997). de Juan-Sanz et al. (2017) found evidence for Ca^2+^ release from the ER in response to a single AP or a train of 20 action potentials evoked at 20 Hz. However, recently been shown that Ca^2+^ release from the ER contributes to train-evoked Ca^2+^ elevation in pyramidal neurons (Tran and Stricker, 2021). As demonstrated in this article, the depletion of the caffeine-sensitive Ca^2+^ stores did not alter the amplitude of depolarization-induced Ca^2+^ transients evoked by low-frequency activity. However, long 5 s KCl depolarization or long train EFS (50 pulses, 9 Hz) of CA1 pyramidal cells induced Ca^2+^ release not only from dendritic ryanodine-sensitive Ca^2+^ stores, which was shown previously (Alford et al., 1993; Garaschuk et al., 1997; Tran and Stricker, 2021) but also at sub-membrane and central regions. They suggested that RyR-mediated Ca^2+^ release from presynaptic intracellular stores contributes to the activation of downstream Ca^2+^- dependent pathways and signaling molecules, including CaMKII, which are required for LTD induction (Arias-Cavieres et al., 2018). Also, we show that the Ca^2+^ signal at the nucleus significantly depends on Ca^2+^ release from the central ER.

Neuronal dendrites play dominant roles in signal integration, neural computation, plasticity, and structurally associated adaptability (Ashhad and Narayanan, 2019). Ca^2+^ influx from the outside *via* voltage- and ligand-gated ion channels, specific synaptic activation can cause the recruitment of intracellular Ca^2+^ stores. Its results in Ca^2+^ release from the ER *via* intracellular Ca^2+^ release channels (IP_3_R and RyR) in dendrites (Nakamura et al., 1999). In the present study, we have shown that calcium signals in the dendritic tree appear faster than in other regions, but the peak value of Ca^2+^ transients in this area is significantly lower than inside the soma. These differences can be explained by the fact that the diameter of the dendrites is ~1 μm, and the calcium signal in it propagates over a shorter distance, in contrast to the soma, and with a smaller dendrite Ca^2+^ release from the ER. The diffusion of calcium inside neurons is determined not only by distance but also by the intracellular binding sites that rapidly bind free calcium ions (Matthews and Dietrich, 2015). The interplay of several systems that release Ca^2+^ into the cytosol and multiple mechanisms that buffer Ca^2+^ in the cytosol sequester Ca^2+^ in intracellular organelles and extrude Ca^2+^ across the plasmalemma provide the necessary control (Blaustein, 1988) that could be different in sub-membrane, central or dendritic tree space of the neuron.

Spontaneous, localized Ca^2+^ release events are found in cardiac myocytes [Ca^2+^ sparks; (Cheng et al., 1993)] and also reported in dendrites of hippocampal pyramidal neurons (Manita and Ross, 2009; Miyazaki and Ross, 2013). In cardiac myocytes and skeletal muscle, depolarization activates voltage-gated L-type Ca^2+^ channels in the surface membrane resulting in localized, sub-membrane increases in cytosolic Ca^2+^. Individual Ca^2+^ release units or clusters of RyRs (Stern et al., 1999) are activated essentially simultaneously. The spatial and temporal summation of Ca^2+^ release from individual Ca^2+^ release units gives rise to whole-cell Ca^2+^ transients (Cheng et al., 1994; Hüser et al., 1996; Shkryl and Blatter, 2013). In skeletal fibers and cardiac ventricular myocytes, the extensive transverse (t) tubular network exists that assures physical proximity of surface membrane Ca^2+^ channels and clusters of RyRs in the SR throughout the entire cell volume (Soeller and Cannell, 1999). It ensures highly synchronized and spatially rather homogeneous RyRs-based Ca^2+^ release from the SR (Cannell et al., 1994; Shkryl and Blatter, 2013). In contrast, atrial cells have poorly developed or even entirely lacking t-tubular system (Hüser et al., 1996; Mackenzie et al., 2001; Smyrnias et al., 2010). Due to these ultrastructural arrangements, action potential (AP)-induced membrane depolarization activates Ca^2+^ entry through VGCCs and CICR through RyRs in the sub-membrane region. Elevation of peripheral [Ca^2+^]_i_ propagates *via* CICR in a Ca^2+^ wave-like fashion in centripetal direction by a diffusion-reaction process (Keizer et al., 1998; Shkryl and Blatter, 2013; Blatter, 2017). In pyramidal neurons studied here, the Ca^2+^ response to APs was likely similar to the one observed in atrial myocytes (Shkryl and Blatter, 2013; Blatter, 2017). The presence of not only RyRs but also of the high density of IP_3_ receptors in hippocampal neurons (Verkhratsky and Shmigol, 1996; Berridge, 1998; Nakamura et al., 2000) makes this process more complicated, requiring not only calcium-mediated activation of RyRs in central ER but also activation IP_3_Rs through the diffusion of IP_3_ from the periphery cell to the central ER to activate IP_3_ receptors in none sub-membrane ER (Blatter et al., 2021); and glutamate-mediated synaptic transmission could contribute a notional part of EFS-induced Ca^2+^ transient that IP_3_ receptors could mediate part of the response. In experiments on freshly isolated hippocampal neurons (data not shown), the amplitude of calcium responses was significantly lower during EFS but did not differ during depolarization of solutions with a high KCl, which suggests the role of glutamate receptors in these responses.

Some limitations are present in the study. Caffeine in high concentration could chelate calcium in the lumen of the ER (Rojo-Ruiz et al., 2018) interfering with RyRs activity and reduce the caffeine-induced Ca^2+^ signal. Caffeine interacts with fluorescent calcium indicator dyes (Muschol et al., 1999). In experiments using one wavelength mode excitation, it affects the response amplitude. However, in the ratiometric mode, it is eliminated by the ratiometric. Some studies show that the Fura-2 dye limits the correct recording of Ca^2+^ (Bootman et al., 2013; Shkryl, 2020) or could be saturated with a high Ca^2+^ response, which creates an additional error in determining the actual value of the calcium concentration. Using single wavelength dye like Fluo-4 with a higher ratio of maximal to minimal fluoresce with more precision and the ability to record small Ca^2+^ events like sparks. Limitations of temporal and spatial resolution reduced the ability to reveal more detailed data on Ca^2+^ signaling in pyramidal hippocampal neurons, and fast 2D or 3D confocal microscopy is required. However, the ratiometric measurements of Fura-2 loaded neurons made it possible to achieve the required accuracy of a given signal in various areas of an individual neuron.

In summary, we demonstrate spatial dynamics of free Ca^2+^ concentration in individual rat hippocampal pyramidal neurons in culture recorded by 2D ratiometric fluorescence of Fura-2 signals. 2D imaging of Ca^2+^ transients revealed an asynchronous delayed rise of free Ca^2+^ concentration in the central area compared to near-membrane or dendritic tree regions. The nuclear response was delayed respectively to other cellular parts. Activation of ryanodine receptors by caffeine triggered a rapid rise of [Ca^2+^]_i_ in all four analyzed regions of interest. Using the RyR agonist caffeine as a pharmacological tool, we found that functional Ca^2+^ stores resize in the somata and peripheral and dendrites of CA1 pyramidal cells. The treatment with caffeine significantly reduces the peak amplitude of Ca^2+^ transients induced by the KCl depolarizing solution. Short electrical stimulation (1 s) was not enough to enhance the Ca^2+^ signal through CICR, but adequate at long-lasting excitation (6 s) produce calcium release from the ER store there 30% of the peak amplitude of Ca^2+^ transient corresponds to RyRs mediated calcium release.

## Data Availability Statement

The raw data supporting the conclusions of this article will be made available by the authors, without undue reservation.

## Ethics Statement

All experimental procedures were performed following ethical principles of the European Convention for the protection of vertebrate animals used for experimental and other scientific purposes (86/609/EEC; European convention, Strasburg, 1986) and were approved by the local Animal Ethics Committee of the Bogomoletz Institute of Physiology (Kyiv, Ukraine).

## Author Contributions

VS carried out the experiment; conceived and designed the analyses; collected the data; performed the analysis; wrote the article.
